# Spigelian hernia: a multi-site review of operative outcomes of surgical repair in the adult population

**DOI:** 10.1007/s10029-023-02946-1

**Published:** 2024-01-23

**Authors:** C. R. Clark, M. L. Kelly, P. Palamuthusingam

**Affiliations:** 1 Department of General Surgery, Royal Brisbane and Women’s Hospital, Brisbane, QLD 4029 Australia; 2 Department of General Surgery, Townsville University Hospital, Townsville, QLD 4814 Australia; 3 Department of General Surgery, Mater Hospital Townsville, Townsville, QLD 4812 Australia; 4 James Cook University, Townsville, QLD Australia

**Keywords:** Spigelian, Hernia, Open, Laparoscopic, Repair, Abdominal

## Abstract

**Purpose:**

Spigelian hernias arise at the linear semilunaris and account for approximately 1–2% of abdominal hernias. The aetiology is due to a defect of the aponeurosis of the transverse abdominis and when discovered, management is surgical intervention. The aim of this study was to observe operative outcomes for open and minimally invasive repair.

**Methods:**

A retrospective chart review was conducted at two hospitals in Townsville, The Townsville University Hospital and The Mater Private Hospital over a 10-year period (2010 to 2020). A surgical database search (ORMIS & IEMR) was performed at both locations using key search terms, including “spigelian hernia”, “laparoscopic”, “open”. Descriptive statistics were utilised to analyse patient factors and operative outcomes in the public and private setting.

**Results:**

43 cases of Spigelian hernias (25 female, 18 male) were reported over the study period. The average age was 66. There were 36 elective cases and 7 emergency cases. A laparoscopic approach was the preferred method of repair, occurring in 74% of cases. Of these cases, the predominant hernial content was fat only. 65% of cases had a history of prior abdominal surgery unrelated to the “Spigelian belt” location. Complications occurred in 19% of cases. Other variables, such as ethnicity, smoking status, defect size, predisposing factors and recurrence rate, were analysed and did not yield statistical significance.

**Conclusion:**

Although a small sample size, the data suggest there is no statistically significant difference between operative outcomes, complication rate and predisposing factors between open and minimally invasive case groups.

## Introduction

**Fig. 1:**
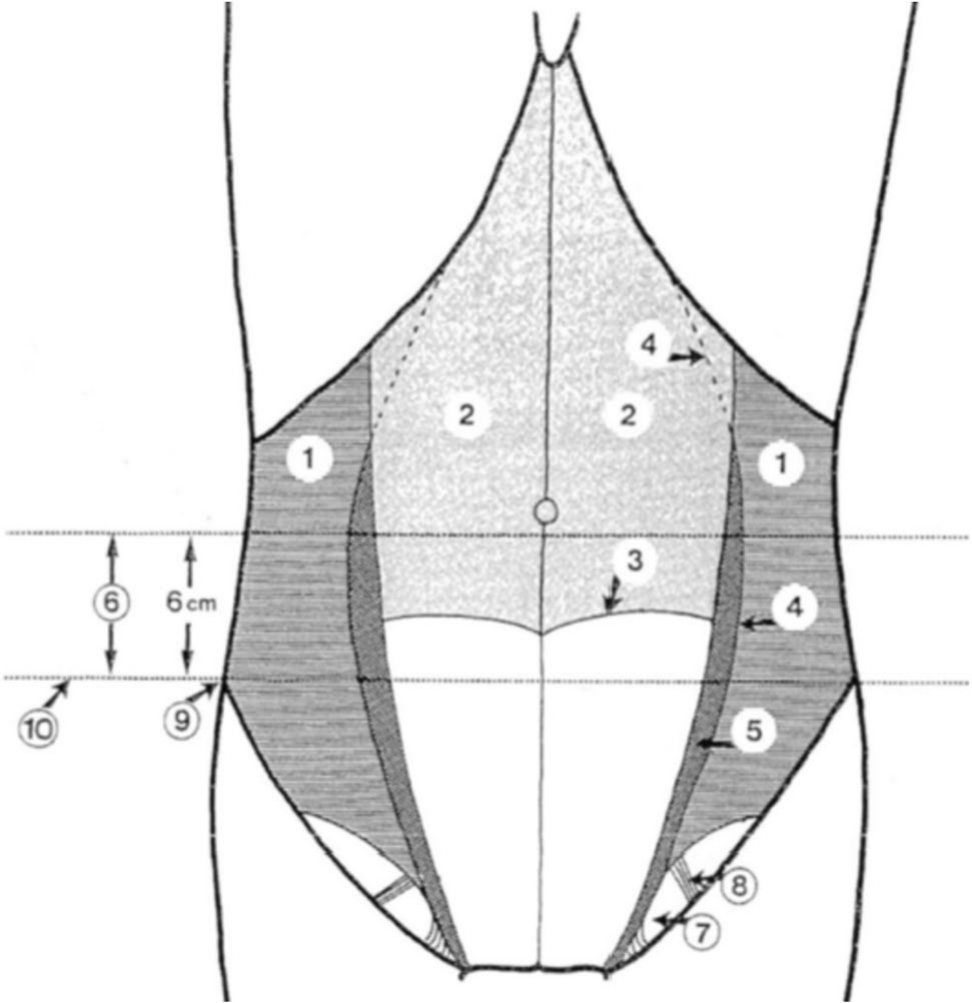
Diagrammatic representation of posterior view of the anterior abdominal wall. (1) Transversus abdominis muscle, (2) Dorsal lamella of the rectus sheath, (3) Arcuate line, (4) Semilunar line, (5) Spigelian point, (6) Spigelian hernia belt, (7) Hesselbach’s Triangle, (8) Inferior epigastric vessels, (9) Anterior superior iliac spine, (10) Interspinal plane [2]

Spigelian hernias, also known as lateral ventral hernias, are a rare type of abdominal hernia arising at the linear semilunaris through a defect of the aponeurosis of the transversus abdominis muscle [1, 2]. This occurs most commonly within a 6 cm wide transverse zone located above the inter-spinal plane known as the ‘Spigelian hernia belt’ (Fig. 1) [1, 2]. Within this zone, the aponeurosis is at its widest and the fibres of the transversus abdominis and internal oblique muscles run parallel to each other, thus creating a weak region that is vulnerable to separation and subsequent hernia formation [2, 3]. The most vulnerable portion is at the intersection of the semilunar line and the arcuate line [2].

Accounting for approximately 1–2% of all abdominal hernias, spigelian hernias are either congenital or acquired [2, 3]. Congenital cases can be associated with ipsilateral undescended testis or cryptorchidism. They are extremely rare, occurring exclusively in the paediatric population, with only 71 reported cases in English language literature since 1946 [2, 4, 6–8]. Acquired cases most commonly occur in an older female population and result from deterioration of the integrity of the abdominal wall and/or chronically raised intra-abdominal pressure, often secondary to chronic obstructive pulmonary disease (COPD), ageing, collagen disorders, pregnancy, obesity or significant weight loss [2–4].

Due to the difficulty of diagnosis, the rarity and the high rate of incarceration (27% of all cases), Spigelian hernias are of particular clinical concern [2, 4, 5, 9–11]. Clinical signs and symptoms are often vague and ill-defined, with many cases being detected incidentally or presenting with undifferentiated, intermittent abdominal pain [9]. Only a small portion of patients have examination findings of a palpable, tender abdominal mass [2, 11]. This is because a Spigelian hernia characteristically only penetrates the aponeurosis of the transversus abdominis muscle and internal oblique muscles and not the overlying, thicker aponeurosis of the external oblique muscle [2, 4]. Thus, the hernial sac may expand in the space between the internal and external oblique muscles, preventing the formation of a characteristic palpable hernial mass [2, 4]. Given the ambiguity of its presentation and the rarity of the condition, clinicians may have difficulty with definitive diagnosis [11, 12]. This is often compounded by the unreliability of imaging for diagnosis, as intermittent reduction can occur with positional changes.

Current management of Spigelian hernias is surgical intervention. Traditionally, this is via either an open or a laparoscopic approach [12]. However, the recent advent of robotic technology introduces another alternative approach. Whilst there have been multiple studies comparing laparoscopic and conventional open techniques for repair of midline ventral hernias, there is still limited evidence on Spigelian hernia repair [9–12].

## Materials and methods

### Ethics

Ethics approval was granted by the Townsville Hospital and Health Service Human Research Ethics Committee (EC00183). Site approval was then individually sought from both Townsville University Hospital and The Mater Private Hospital, Townsville.

### Study design

A retrospective data review was conducted at two major hospitals in the regional town of Townsville, Queensland; Townsville University Hospital and The Mater Private Hospital. Electronic and paper-based clinical records were reviewed from a 10-year period of 2010 until 2020 to identify patients that had been diagnosed with a Spigelian hernia. Inclusion criteria composed of adult patients (18 years and older) who had documented operative management and a documented post-operative follow-up visit.

Patient demographics and clinical outcomes data, including age, gender, predisposing factors and contributing factors were collected for each identified patient. Predisposing factors were defined as conditions resulting in chronically raised intra-abdominal pressure, chronic obstructive pulmonary disease (COPD), advancing age, collagen disorders, obesity and recent significant weight loss. Contributing factors were defined as precipitating heavy lifting and smoking.

Operative reports were reviewed for information pertaining to operative factors and complications, including: surgical approach (open, laparoscopic, robotic), method of repair (utilisation of sutures versus mesh), defect size, hernial sac contents, wound dehiscence, infection, chronic pain, haematoma formation, seroma formation, mesh erosion, adherence to post-operative advice, length of hospital stay and recurrence. Complications occurring within three months post-operatively were recorded and graded according to the Clavien–Dindo Classification Criteria. The urgency of the repair was recorded in order to assess whether elective repair versus emergency repair had a significant impact on operative factors, complication occurrence and overall morbidity.

Excluded patients included those with incisional hernias misdiagnosed as Spigelian hernias, paediatric patients and hernias of uncertain diagnosis.

### Data analysis and statistics

Due to the small sample size, no in-depth statistical analysis was deemed appropriate. All results are presented as descriptive statistics. Basic descriptive statistics are presented as mean, medians and ranges for continuous variables and percentages for categorical variables, unless otherwise stated.

## Results

43 individuals (18 male, 25 female) were identified to have been diagnosed with a Spigelian hernia and underwent operative management during the selected study period. Table 1 outlines the demographical characteristics of the dataset. The average age was 66 years old with an age range of 30 to 98 years. 95% of participants were of Caucasian descent and only 5% of individuals were identified as of Aboriginal or Torres Strait Islander descent. 58% of participants received care in the public setting, whilst 42% received care in the private setting. No patients underwent open repair in the private setting.

**Table 1:** Demographical data

demographic data	Open repair (*n* = 11)	Laparoscopic repair (*n* = 32)	Total (*n* = 43)
Age (years)			
18–24	0	0	0
25–34	0	2	2
35–44	1	3	4
45–54	1	3	4
55–64	2	5	7
65–74	6	7	13
75 and above	1	12	13
Gender			
Female	8	17	25
Male	3	15	18
Ethnicity			
Aboriginal and torres strait islander	0	2	2
Caucasian	11	30	41
BMI			
< 18	0	0	0
18–24	2	2	4
25–30	2	4	6
> 30	4	6	10
Unknown	3	20	23
Smoking status			
Smoker*	3	4	7
Non-smoker	8	28	36
Predisposing factors**			
Present	10	23	33
Absent	1	9	10
Precipitating heavy lifting			
Yes	0	8	8
No	11	24	35
Previous Abdominal Surgery			
Yes	8	20	28
No	3	12	15
Previous pregnancy			
Yes	5	6	11
No	5	17	22
Unknown	1	9	10
Previous abdominal hernia			
0	8	21	29
1	2	7	9
2	0	3	3
3 or more	1	1	2
Hospital Setting			
Public	11	14	25
Private	0	18	18

^*^ smoker defined as current smoker or participant who has quit smoking < 10 years ago

^**^ predisposing factors: chronically raised intra-abdominal pressure, COPD, ageing, collagen disorders, pregnancy, obesity, recent significant weight loss

The BMI data were available for 23 patients and ranged from 18 to 41, with the average BMI identified as 31. Ten out of 20 individuals were identified to have a BMI of > 30. Furthermore, 77% of participants suffered from a predisposing factor, the most common of which were obesity and advancing age. It should be noted that although collagen disorders and recent significant weight loss were identified in the literature as predisposing factors, none of the participants in this study had a recorded history of suffering from either of these conditions.

19% of participants reported heavy lifting as a precipitant event and 16% of participants were current smokers or smokers that had ceased smoking within the last 10 years. 65% of participants reported previous abdominal surgery of which ventral hernia repair, laparotomy, laparoscopic bowel resection, hysterectomy, Hartman’s Procedure, Lower Segment Caesarean Section and Transurethral Resection of the Prostate, were the most common. 33% reported previously suffering from an abdominal hernia. Of the 14 cases of previous abdominal hernia, 2 participants had previously suffered from a Spigelian hernia. Patients undergoing previous abdominal surgery or hernia repair less than 6 months from their presentation with a Spigelian hernia were excluded from the dataset. In this way, potential incisional hernias were not included in our study.

Table 2 demonstrates the operative setting and surgical approaches pertaining to each surgical encounter. Overall, 84% of participants were symptomatic immediately prior to surgical intervention. 84% of cases were performed in the elective setting and 16% were emergencies. No emergency cases were performed privately. In the public setting, 40% of cases were performed open and 56% were laparoscopic. In the private setting, 100% of cases were performed laparoscopically. There were no cases performed via a robotic approach at either facilities.

**Table 2:** Operative factors

Operative Factor	Public Setting (*n* = 25)	Private Setting (*n* = 18)	Total (*n* = 43)
Symtomatology			
Symptomatic	22	14	36
Asymptomatic	3	4	7
Affected side			
Left	11	10	21
Right	14	7	21
Bilateral	0	1	1
Operative approach			
Open	10	0	10
Laparoscopic	14	18	32
Laparoscopic converted to open*	1	0	1
Concurrent procedure			
Performed	7	9	16
Not performed	18	9	27
Method of repair			
Mesh	20	4	24
Sutures	5	14	19
Urgency of case			
Elective setting	18	14	36
Emergency setting	7	18	7

^*^smoker defined as current smoker or participant who has quit smoking < 10 years ago

^**^predisposing factors: chronically raised intra-abdominal pressure, COPD, ageing, collagen disorders, pregnancy, obesity, recent significant weight loss

Mesh was utilised in 56% of cases (80% public versus 22% private) and sutures were utilised in 44% of cases (20% public versus 78% private). 37% of repairs occurred concurrently with another surgical intervention, such as adhesiolysis, other hernia repair (abdominal, inguinal, etc.) or appendicectomy.

The type of mesh/suture utilised for repairs and the layers of the repair material were placed in can be found outlined in Table 3. Overall, the most commonly used mesh was Physiomesh (ETHICON, Johnson & Johnson), utilised in 33% of all mesh repairs (8 out of 24). The most commonly used suture material was PDS sutures, utilised in 11% of all suture repairs (2 out of 19). It was found that the specific material of repair utilised was omitted from the operative notes of 19 participants in total. Of the cases performed publicly, conventional open suture repair technique was the most common repair method for suture repairs. Pre-peritoneal sublay mesh repair was the most common method of mesh repair. Privately, laparoscopic suture technique and trans-abdominal pre-peritoneal repair (TAPP) were the most commonly utilised methods of repair for suture and mesh repairs, respectively.

**Table 3:** Hernia repair factors

Hernial repair factor	Public setting (*n* = 25)	Private setting (*n* = 18)	Total (*n* = 43)
Mesh type			
Ultrapro (ETHICON, Johnson & Johnson, USA)	3	0	3
Ventralex ST mesh (BARD, Becton, Dickson and Company, USA)	2	0	2
Prolene mesh (ETHICON, Johnson & Johnson, USA)	3	0	3
3DMax Light Mesh (BARD, Becton, Dickson and Company, USA)	1	0	1
Physiomesh (ETHICON, Johnson & Johnson, USA)	8	0	8
Vicryl mesh (COVIDIEN, USA)	1	0	1
LP Bard Composix L/P Mesh (BARD, Becton, Dickson and Company, USA)	1	0	1
C-QUR mesh (Atrium, New Hampshire, USA)	0	1	1
Not Stated	1	3	4
Suture type			
PDS	2	0	2
Nylon	1	0	1
Maxon	1	0	1
Vicryl	1	0	1
Not Stated	0	14	14
Layer/Type of repair			
Total Extraperitoneal Repair (TEP)	6	0	6
Transabdominal Preperitonal Repair (TAPP)	8	1	9
Preperitoneal Onlay	0	0	0
Preperitoneal Inlay	1	0	1
Preperitoneal Sublay	5	0	5
Intraperitoneal Sublay	0	0	0
Open Suture	4	0	4
Laparoscopic Suture	1	14	15
Not Stated	0	3	3

Operative outcomes are presented in Table 4. 65% of cases contained only fat in the hernial sac at the time of operation. 16% of cases contained omentum and 19% contained visceral organs. A similar proportion of open repairs contained organ content when compared with laparoscopic repairs (18% and 19% respectively). Hernia defect size was graded according to the European Hernia Society (EHS) primary hernia classification system [13]. Of the 23 cases where hernial defect size was recorded, only 8.7% of participants were found to have a hernial defect size of greater than 4 cm. One additional case performed in the public setting reported a hernial defect of significant size, requiring drain placement at the time of operation.

**Table 4:** Operative outcomes

Operative Outcome	Open Repair (*n* = 11)	Laparoscopic Repair (*n* = 32)	Total (*n* = 43)
Contents of Hernial Sac			
Fat containing only	6	22	28
Omentum containing	3	4	7
Organ containing	2	6	8
Defect size (cm)			
EHS Small (< 2)	7	6	13
EHS Medium (2–4)	1	7	8
EHS Large (> 4)	0	2	2
Not recorded	3	17	20
Length of Hospital Stay (days)			
1	6	24	30
02-May	4	8	12
> 5	1	0	1
Complications			
Yes	4	4	8
No	7	28	35
Clavien Dindo Score			
0	7	28	35
1	2	3	5
2	0	1	1
3	2	0	2
4	0	0	0
5	0	0	0
Follow Up (days)			
< 30	4	21	25
30–60	5	6	11
> 60	2	5	7
Adherence to post-operative advice*			
Yes	10	31	41
No	1	1	2
Recurrence			
Yes	2	0	2
No	9	32	41

^*^adherence to post-operative advice: compliance with no heavy lifting for 6 weeks post-operatively

Note: 14 senior surgeons contributed to the case load across the two facilities

The median length of hospital stay was one day. Length of stay overall ranged from one to seven (1–7) days. There was a longer length of stay in patients undergoing open repair compared to laparoscopic repair (45% compared with 25%).

Complications occurred in 19% of cases, with open repair patients experiencing more complications compared to the laparoscopic counterparts (36% versus 13% respectively). No participant experienced a complication in keeping with a Clavien–Dindo score of 4 or 5. Common complications in the dataset included seroma formation (7% of cases), superficial wound infection (7% of cases) and haematoma formation (5% of cases). There were no reported incidences of chronic pain or mesh erosion. Of those that scored a 2 or higher, the complications included aspiration at induction of anaesthesia, allergic reaction to antibiotics, wound dehiscence around a drain site and post-operative ileus. In the subset of participants experiencing a Clavien–Dindo complication score of 2 or more, the predominant operative approach was open repair (67%). In cases where complications occurred that may be attributed to surgical factors, the materials implicated included: PDS suture (1 case), (ETHICON, Johnson & Johnson) Prolene mesh (2 cases) and not stated (3 cases).

Adherence to post-operative advice was defined as patient compliance with instructions to avoid heavy lifting > 5 kg for 6 weeks immediately post-operatively. Two patients subjectively reported non-adherence to this advice. Of these two patients, one experienced a hernial recurrence. Ultrapro mesh (ETHICON, Johnson & Johnson) was the material utilised in this case. An additional one patient who did adhere to post-operative advice was recorded to have a hernial recurrence at 42 days. This occurred in the participant requiring drain placement at time of operation, and prolene mesh (ETHICON, Johnson & Johnson) was utilised. Both cases of recurrence occurred in open repairs where mesh was the method of repair utilised.

Follow-up ranged from 10 to 365 days. A total of 5 outliers were identified where follow-up was continued until 150–365 days. In the context of these outliers, the mean follow-up was 56 days and the median follow-up was 25 days.

## Discussion

The authors present the first study analysing operative and patient outcomes of Spigelian hernia repair in the clinical settings of private and public hospital systems, as well as in both elective and emergency environments. The current landmark study on Spigelian hernia repair in the adult population was undertaken by Moreno-Egea et al. in 2002, who found that there was no statistically significant difference between laparoscopic and open approaches for epidemiological and diagnostic factors. However, they identified that laparoscopy was associated with statistically proven advantages for morbidity and length of hospital stay [9]. The results of this study emulate these findings and henceforth represent a contemporary update on the surgical management of Spigelian hernias in a more diverse clinical setting. Consistent with the wider literature, the results of the study also showcase how the field of general surgery is leaning towards a minimally invasive approach due to its association with shorter length of stay, lower complications and reduced rates of recurrence.

Through scrutinising Spigelian hernia repair in both a public and private setting as well as in emergency and elective environments, this study provides a unique, overarching review of Spigelian hernias. It is the first study to account for the variability of patient and operative outcomes that may arise when the setting of presentation and operation are differing. Interestingly, no emergency or open cases were performed in the private setting. This may reflect the inherent nature of private practice, where most private hospitals do not have the capacity to offer 24-h emergency surgical cover and may not have the capacity to treat surgically complex individuals who potentially warrant an open approach. It may also serve to reiterate the shift of General Surgery practice to a minimally invasive approach when it is safe to do so. Due to the unavailability of robotic technology in Townsville Queensland in the study period, no robotic Spigelian hernia repairs were observed.

This study is not without limitations, given the small sample size and uneven distribution of case groups. A rudimentary first look may reveal that the open approach was associated with higher complication rates; yet these complications were largely composed of anaesthetic complications and drug reactions, and not poor surgical outcomes. Given these exceptions, no statistically significant difference could be observed. Furthermore, prolene mesh (ETHICON, Johnson & Johnson) was implicated in 2 cases where complications could be attributed to surgical factors and 1 case of recurrence. Additional research is required on the utilisation of prolene mesh compared to other mesh types in Spigelian hernia repair specifically, to more holistically understand its comparative performance.

The researchers also identified issues with data recording, whereby vital facts surrounding the presentation, nature and material of repair were omitted from a number of medical records. The move towards establishing large volume databases for operative procedures encourages the development and use of more structured operative surgical records to allow extraction of more consistent data.

Finally, large inconsistencies were noted regarding the follow-up of patients included in the study. Follow-up ranged from 10 to 365 days. Unfortunately, a large proportion of participants were therefore followed up for an inadequate period of time post-operatively. This is a major criticism of this study. However, it clearly accentuates the need for implementation of a standardised approach to hernia management, which includes guidelines regarding appropriate follow-up time frames.

A larger study is required to gain a holistic understanding of the patient factors and operative outcomes associated with open and minimally invasive surgical repair. Future ventures on Spigelian hernias could involve expansion of this study to include multiple other centres across Queensland, to further assess operative outcomes and improve patient management. Nonetheless, the results of this study set to build on evidence with a larger dataset in both private and public settings on operative outcomes in Spigelian hernias. It showcased that the current operative outcomes of a rare general surgical ailment managed in a regional centre mirror the outcomes achieved in high-volume centres, as described in available literature.

## Conclusion

Spigelian hernias are a rare form of abdominal hernia arising at the linear semilunaris. Due to the difficulty of diagnosis and the high rates of incarceration associated with their occurrence, surgical intervention is currently the only appropriate management. This study found no statistically significant difference between operative outcomes, complication rate and predisposing factors when comparing open and minimally invasive case groups. The results of the study support existing literature, with a minimally invasive approach associated with marginally superior morbidity and length of hospital stay, offering patients advantageous outcomes. Future advances/studies should include more powered research on this rare surgical ailment to inform clinicians about best practice management of Spigelian hernias.
